# Rab11-FIP3 is a cell cycle-regulated phosphoprotein

**DOI:** 10.1186/1471-2121-13-4

**Published:** 2012-03-08

**Authors:** Louise L Collins, Glenn Simon, Johanne Matheson, Christine Wu, M Clare Miller, Tetsuhisa Otani, Xinzi Yu, Shigeo Hayashi, Rytis Prekeris, Gwyn W Gould

**Affiliations:** 1 Henry Wellcome Laboratory of Cell Biology, Institute of Molecular, Cell and Systems Biology, College of Medical, Veterinary and Life Sciences, Davidson Building, University of Glasgow, Glasgow G12 8QQ, Scotland, UK; 2 Department of Cell and Developmental Biology, University of Colorado Anschutz Medical Campus, 12801 E. 17th Avenue, Aurora, CO 80045, USA; 3 Department of Cell Biology and Physiology, University of Pittsburgh Medical School, Pittsburgh, PA 15260, USA; 4 Riken Centre for Developmental Biology, 2-2-3 Minatojima-minamimachi, Chuo-ku Kobe 650-0047, Japan

**Keywords:** Cytokinesis, Rab11-FIP3, Cdk1, Endosomes

## Abstract

**Background:**

Rab11 and its effector molecule, Rab11-FIP3 (FIP3), associate with recycling endosomes and traffic into the furrow and midbody of cells during cytokinesis. FIP3 also controls recycling endosome distribution during interphase. Here, we examine whether phosphorylation of FIP3 is involved in these activities.

**Results:**

We identify four sites of phosphorylation of FIP3 in vivo, S-102, S-280, S-347 and S-450 and identify S-102 as a target for Cdk1-cyclin B *in vitro*. Of these, we show that S-102 is phosphorylated in metaphase and is dephosphorylated as cells enter telophase. Over-expression of FIP3-S102D increased the frequency of binucleate cells consistent with a role for this phospho-acceptor site in cytokinesis. Mutation of S-280, S-347 or S-450 or other previously identified phospho-acceptor sites (S-488, S-538, S-647 and S-648) was without effect on binucleate cell formation and did not modulate the distribution of FIP3 during the cell cycle. In an attempt to identify a functional role for FIP3 phosphorylation, we report that the change in FIP3 distribution from cytosolic to membrane-associated observed during progression from anaphase to telophase is accompanied by a concomitant dephosphorylation of FIP3. However, the phospho-acceptor sites identified here did not control this change in distribution.

**Conclusions:**

Our data thus identify FIP3 as a cell cycle regulated phosphoprotein and suggest dephosphorylation of FIP3 accompanies its translocation from the cytosol to membranes during telophase. S102 is dephosphorylated during telophase; mutation of S102 exerts a modest effect on cytokinesis. Finally, we show that de/phosphorylation of the phospho-acceptor sites identified here (S-102, S-280, S-347 and S-450) is not required for the spatial control of recycling endosome distribution or function.

## Background

Membrane traffic to the furrow is an essential facet of cytokinesis [1-5]. The plasma membrane of the furrow of dividing cells has a distinct lipid and protein composition as compared to the rest of the plasma membrane, with the furrow enriched in cholesterol and phosphatidylinositol 4, 5-bisphosphate (for example) and various proteins involved in intracellular membrane trafficking (e.g. dynamin, SNAREs, etc.) [2,5,6]. Exocytosis of intracellular membranes occurs to the furrow and intercellular bridge, reflecting a requirement for the delivery of intracellular signalling and/or membrane re-modelling activities to the correct spatial co-ordinates during abscission [1-4]. Identifying the molecular basis of these trafficking events is crucial for a full understanding of cell division.

We previously have shown that Rab11 and its effector molecule, Rab11-FIP3 (FIP3), associate with recycling endosomes and traffic into the furrow and midbody [7,8]. Depletion of FIP3 or expression of a FIP3 mutant unable to bind Rab11 results in defective abscission, and both these genes are over-expressed in cancer [5]. FIP3 can bind both Rab11 and Arf6 GTPases simultaneously, and FIP3, Rab11 and Arf6 form a ternary complex *in vitro *[9], prompting us to suggest that multiple interactions between Rab11 and Arf6 with the Exocyst complex may serve to anchor FIP3-containing vesicles in the midbody prior to abscission [9], where they function as an organisation platform for the assembly of the abscission machinery [10]. Recent studies have also suggested that a dynamic interaction of FIP3 with the centralspindlin component Cyk-4 may also contribute to the localisation of FIP3 during mitosis [11]. FIP3 exhibits profound spatial and temporal dynamics during cell division [8,9]. GFP-FIP3 redistributes from diffuse cytosolic staining onto membranes at the centrosome during early anaphase; GFP-FIP3 then begins to accumulate in the developing furrow before entering the midbody and at very late telophase accumulating at or close to the midbody ring. Similar distributions have recently been reported for endogenous FIP3 [12]. In addition to its role in mitosis, FIP3 has also been suggested to control the spatial distribution of recycling endosomes in interphase cells [7,13], raising the question of how these distinct functions are modulated. Recent work has revealed that FIP3 is a phospho-protein with multiple phospho-acceptor sites [14]. Phosphorylation of residues S-488, S-538, S-647 and S-648 modulates the interaction of FIP3 with motor machinery, thereby controlling the spatial distribution of endosomes [14]. However, there is no published work relating phosphorylation of FIP3 at these or other sites to events in the cell cycle.

Here, we identify and characterise four sites of FIP3 phosphorylation: S-102, S-280, S-347 and S-450. We show that Cdk1, which is active in metaphase [15-17], phosphorylates FIP3 exclusively on S-102 *in vitro*, and that S-102 phosphorylation of FIP3 declines in parallel with cyclin B levels as cells exit metaphase and proceed into telophase. A phospho-mimetic mutation of this site (S102D) exerted a modest effect on cytokinesis. Individual mutants at S-280, S-347 and S-450 or a mutant of FIP3 in which S-488, S-538, S-647 and S-648 were all mutated to alanine had no effect on cytokinesis and did not modulate FIP3 distribution during the cell cycle. We further show that FIP3 is a cell cycle-regulated phosphoprotein and show that the shift in FIP3 distribution from membranes to cytosol occurs concomitantly with dephosphorylation of FIP3, but that dephosphorylation of FIP3 alone is insufficient to promote membrane association *in vitro*. Of the sites identified here, we show that S102 is dephosphorylated as cells move from metaphase into telophase. Finally, we show that phospho-mimetic or phospho-resistant mutants of S-102, S-280, S-347 or S-450 do not modulate transferrin uptake or recycling endosome distribution in interphase cells.

## Results

**Figure 1:**
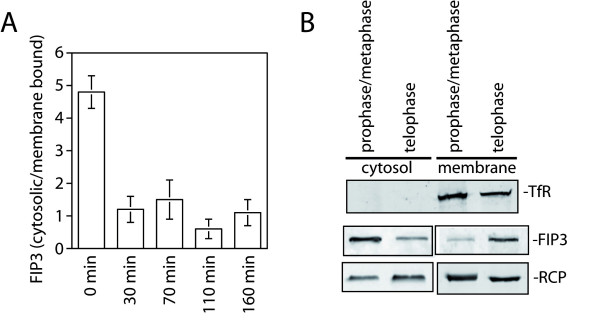
**FIP3 translocates from cytosol to membranes during the cell cycle Panels A and B**. Synchronized HeLa cells were harvested at different time points after release from the nocodazole block as shown. Cells were fractionated into cytosolic and membrane fractions and levels of FIP3 analyzed by immunoblotting, with 60 μg of each fraction loaded in each lane. Data shown in **panel A **are the means and standard deviations from three independent experiments. In **panel B**, "prophase/metaphase" represents samples harvested at 0 min after release from nocodazole block, while "telophase" represents samples harvested 70 min after release from nocodazole block. Transferrin Receptor (TfR) was used as a marker for membranes, and RCP/FIP1 as an unrelated FIP for comparative purposes.

GFP-tagged FIP3 undergoes a re-distribution from a diffuse cytosolic distribution to membrane compartment centred over the centrosome as cells progress from metaphase to anaphase [8]. During early telophase, GFP-FIP3 accumulates near DNA but at the opposite side of the centrosomes, before gradually moving to mid-zone microtubules and then eventually translocating into the midbody in late telophase, just prior to abscission [8,11]. A similar pattern has recently been reported for endogenous FIP3 [12]. We performed a simple subcellular fractionation of HeLa cell homogenates isolated at prophase/metaphase or telophase and studied the distribution of the endogenous protein between membrane and cytosolic fractions (Figure 1A, B). FIP3 was found to re-distribute from the cytosol to membrane fractions in telophase, consistent with previous imaging studies [7-9,12].

**Figure 2:**
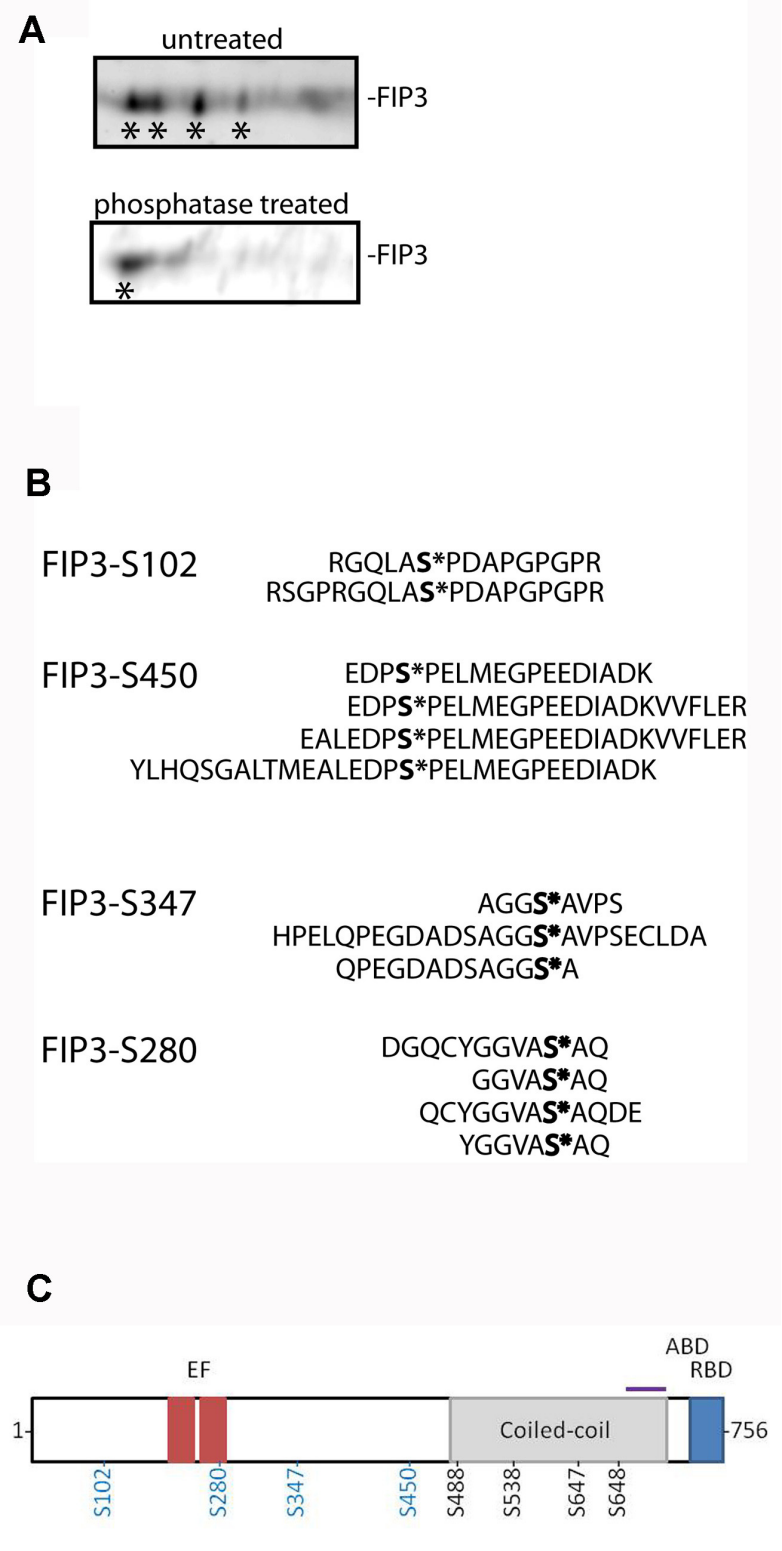
**Identification of putative FIP3 phosphorylation sites Panel A**. Synchronized HeLa cells from metaphase were lysed and FIP3 immuno-precipitated (from untreated or phosphatase-treated lysates) using anti-FIP3 antibodies. Immunoprecipitates were then separated by 2D SDS-Page and immunoblotted with anti-FIP3 antibodies. Asterisks mark different phospho-states of FIP3. A representative blot is shown for each condition. **Panel B**. Synchronized HeLa cells in metaphase were lysed in the presence of phosphatase inhibitors. FIP3 was then precipitated and putative phosphorylation site identified by mass spectrometry. Shown are all the peptides identified for each putative phosphorylation site. **Panel C**. A schematic of FIP3 showing the location of key residues. Phospho-acceptor sites identified here are shown in blue, those in black at the sites mutated to A in the HA-FIP3-4A mutant (see text). The Rab11-binding region (RBD) and Arf6 interaction region (ABD) are shown, together with the approximate position of the EF-hand-like domains and the coiled-coil region.

Previous studies have suggested that FIP3 is a phosphoprotein, with over 20 sites of phosphorylation identified [14]. We hypothesised that phosphorylation/dephosphorylation may represent a potential mechanism for this regulation of FIP3 localisation or function. We immunoprecipitated FIP3 from HeLa cells arrested in metaphase, and examined the distribution of FIP3 on 2D-gel electrophoresis by immunoblotting and compared the signal to that obtained from identical samples treated with alkaline phosphatase (Figure 2A). Phosphatase treatment consistently reduced the number of FIP3-immunoreactive species identified, consistent with the notion that FIP3 is phosphorylated in metaphase. We identified four phospho-peptides by mass spectrometry (Figure 2B), which encompassed S102, S280, S347 and S450 (Figure 2C). Although not all of these sites were observed in the previous study [14], it should be noted that we have focussed our study on FIP3 in metaphase.

### FIP3 is phosphorylated by Cdk1 *in vitro *on S102

**Figure 3:**
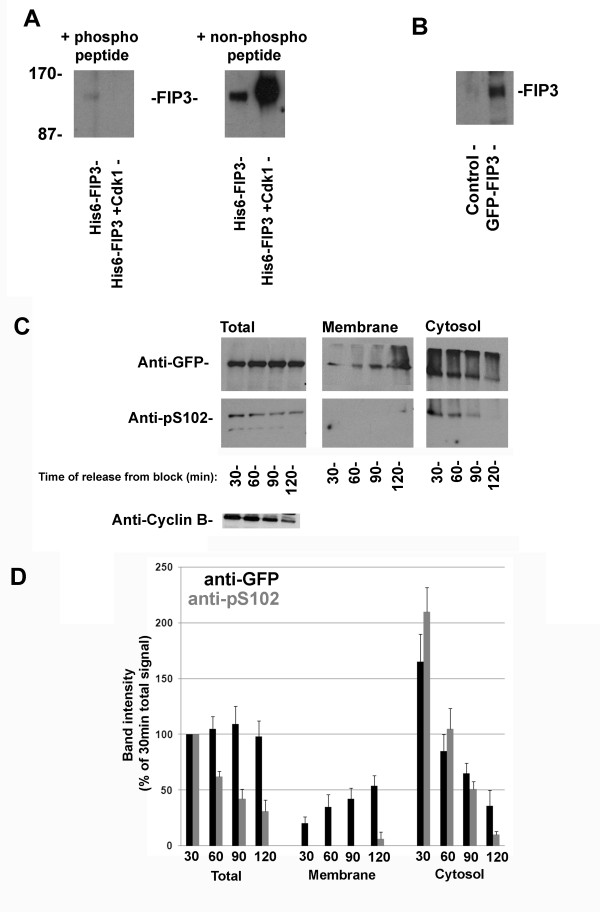
**FIP3 is phosphorylated on S102 in a cell cycle-dependent manner**. **Panel A **200 ng of His_6_-tagged recombinant FIP3, or 200 ng His_6_-tagged recombinant FIP3 incubated with Cdk1-cyclin B to phosphorylate the protein on S102 (see below) were separated by SDS-PAGE, transferred to nitrocellulose and analysed by immunoblotting using 0.2 μg/ml affinity-purified anti-pS102 antibodies, incubated in the presence of either the phosphorylated or non-phosphorylated peptide (2.5 μM) as indicated. The position of molecular weight markers (kDa) is shown. Data from a typical experiment are presented, repeated four times with similar results. **Panel B **HeLa cells expressing GFP-FIP3 (or control cells) were lysed as described in *Methods*. Samples of lysate were separated by SDS-PAGE, transferred to nitrocellulose and probed with anti-pS102 antibodies. GFP-FIP3 is readily detected (endogenous FIP3 is expressed at too low levels to be readily detected by this antibody). **Panel C **HeLa cells stably expressing GFP-FIP3 were synchronised using a thymidine and nocodazole block as described in *Methods*. Cells were harvested 30, 60, 90 or 120 min after release from the block, corresponding to metaphase (~30 min) through late telophase (120 min)[11], fractionated into membrane and cytosolic fractions, and these fractions together with the initial homogenate (total) were immunoblotted with anti-GFP (to reveal total GFP-FIP3 levels in each fraction), anti-pS102 and anti-cyclin B as shown. Data from a typical experiment is presented, repeated three times with quantitatively similar results, which are quantified in **Panel D **(the signals at each point and in each fraction are expressed as a% of that in the 30 minute total cell lysate level. The mean of triplicate experiments (± S.D.) is shown).

We next generated a phospho-selective antibody to detect phosphorylation of residue S102. This reagent readily detected recombinant FIP3 purified from Baculovirus, and also recombinant FIP3 phosphorylated on S102 by incubation with Cdk1-cyclin B (see below); the signal was selectively blocked by the phosphorylated antigenic peptide, but not the corresponding non-phosphorylated control peptide (Figure 3A). This antibody also readily detected phosphorylation of S102 within the context of GFP-FIP3 ectopically expressed in HeLa cells (note that expression of endogenous FIP3 is low, and below the limits of detection with this reagent) (Figure 3B). We found that total levels of phosphorylation of S102 within GFP-FIP3 decreased as cells progressed from metaphase (~30 min) to telophase (~120 min; cf. cyclin B immunoblot) but total cellular levels of GFP-FIP3 (detected with anti-GFP antibodies) were unchanged (Figure 3C, D). Phospho-FIP3 could be detected only within the cytosolic pool of FIP3, and levels of phosphorylation declined markedly as cells progressed from metaphase to telophase (Figure 3C, D).

**Figure 4:**
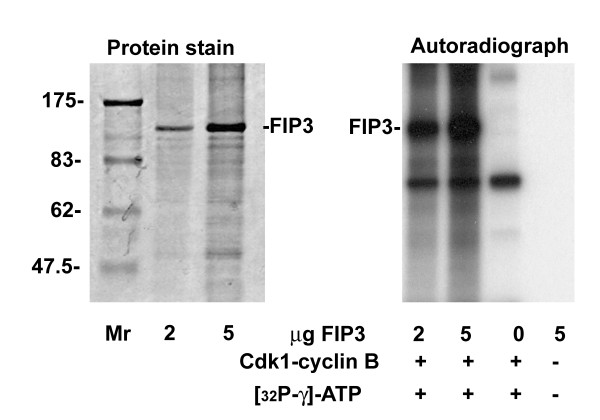
**Cdk1-cyclin B phosphorylates FIP3 on S102**. Recombinant FIP3 was expressed as a His6-fusion protein in Sf9 cells and purified as described in *Methods*. The left panel shows a protein stain of 2 and 5 μg of purified FIP3 with the position of molecular weight markers indicated (kDa). 0, 2 and 5 μg of purified FIP3 were incubated with 50 ng of recombinant Cdk1-cyclin B complex in the presence of ATP-γ-^32^P for 30 min as described (also shown is a control in which 5 μg of FIP3 was incubated without kinase or ATP). Samples were then boiled in Laemmli buffer, separated on SDS-PAGE and examined by autoradiography. Shown is data from a typical experiment, repeated 4 times with similar results.

S102 is within in a consensus sequence similar to that for Proline-directed kinases, such as cyclin-dependent kinase 1 (Cdk1). Consistent with this, Figure 4 shows that recombinant Cdk1-cyclin B readily phosphorylates Baculovirus expressed recombinant FIP3. Mass spectrometry revealed that Cdk1 had phosphorylated recombinant FIP3 only on residue S102 (data not shown, but see also Figure 3A). We also performed *in vitro *kinase assays with Plk1, Aurora A and Aurora B; no specific phosphorylation events could be identified (LLC and GWG, unpublished data). Attempts to generate phospho-specific antibodies to the other sites identified in Figure 2 have thus far proven unsuccessful. Hence, we cannot ascertain whether phosphorylation of S280, S347 or S450 is cell cycle-dependent (but see further below).

### Consequences of FIP3 phosphorylation

**Figure 5:**
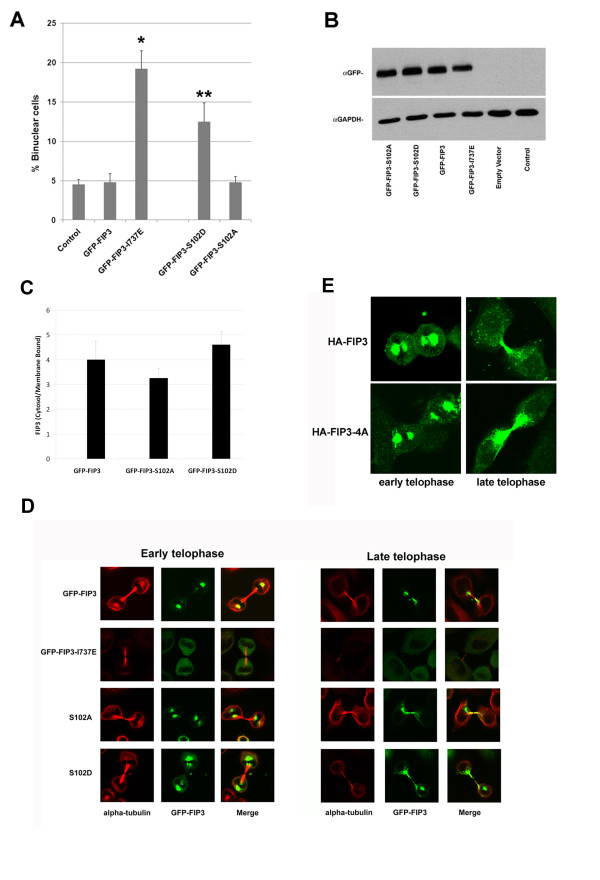
**S102D expression perturbs cytokinesis**. **Panel A **HeLa cells on glass coverslips were transiently transfected with GFP-FIP3, or the indicated mutants as described. After 48 h, cells were fixed and microtubules (anti-tubulin) and DNA (DAPI) stained as described. The fraction of cells expressing each FIP3 species that were binucleate was then determined and is plotted graphically. The data shown are from a representative experiment: in this case over 200 GFP-positive cells were counted per condition. *indicates a significant increase over control (GFP-FIP3) cells p = 0.02, and **p < 0.05. Similar results were obtained in two other experiments of this type, but the fraction of binucleate cells varied between experiments (e.g. the% of binucleate cells expressing GFP-FIP3-I737E varied between 16 and 33%). **Panel B **shows an immunoblot from the same experiment in which equal amounts of lysate were immunoblotted with anti-GFP to show that similar levels of each mutant were expressed. Anti-GAPDH is used as a loading control. **Panel C **shows the cytosol/membrane ratio of the indicated mutants in cells synchronised in metaphase. Data from a representative experiment is shown. **Panel D **HeLa cells on glass coverslips were transiently transfected with GFP-FIP3 (pseudo-coloured green), or the indicated mutants as described. After 24 h, cells were fixed and immunostained with anti-tubulin (pseudo-coloured red) and the distribution of cells in telophase examined. Shown are cells in early or late telophase. Data from a representative experiment, repeated 5 times are shown. Note that the categorisation of these images into 'early' or 'late' telophase is dictated by the appearance of FIP3 (see [11] for details) which is highly dynamic in telophase, moving from a characteristic perientrosomal localisation into the intercellular bridge region as cells move from early to late telophase. **Panel E **HeLa cells expressing HA-FIP3 or HA-FIP3-4A were analysed by immunofluoresence microscopy using monoclonal anti-HA in telophase to reveal the localisation of epitope-tagged FIP3. Shown are typical images of cells in early or late telophase, pseudo-coloured green. Data from a representative experiment, repeated 3 times are shown.

In an attempt to determine the role of phosphorylation of FIP3 at these sites, we generated a panel of either S > A or S > D mutants within the context of GFP-FIP3. Expression of these mutants in HeLa cells (with the exception of S450A - see *Discussion*) did not affect cell growth (data not shown). We next examined whether over-expression of these mutants gave rise to defective cytokinesis by counting binuclear cells. A modest increase in the fraction of binuclear cells was observed in cells expressing GFP-FIP3-S102D. However, this effect was smaller than was observed in cells expressing GFP-FIP3-I737E, a Rab11-binding deficient mutant of FIP3, previously shown to increase the number of binuclear cells [8] despite similar levels of expression of these mutants (Figure 5A, B). Similarly, the membrane/cytosol distribution of GFP-FIP3-S102D revealed a modest decrease in ratio in metaphase compared to GFP-FIP3-S102A or GFP-FIP3 (Figure 5C; compare to Figure 1A). Such data support the possibility that de/phosphorylation of S102 is involved in cell division. Surprisingly, when expressed in HeLa cells as GFP-fusion proteins, the distribution of the individual S > A or S > D mutants of FIP3 appeared indistinguishable from wild-type FIP3 throughout the stages of the cell cycle. Figure 5D shows the localisation in early and late telophase for GFP-S102A and GFP-S102D. Similar results were observed when all other mutants were expressed (data not shown).

### Phosphorylation of ser-488, 538, 647 and 648 is not required for cytokinesis

Recent studies have identified more than 20 phospho-acceptor sites on FIP3 and its fly homologue Nuclear Fallout (Nuf) [14]. Mutation of four phospho-acceptor sites within FIP3 to Alanine (Ser-488, 538, 647 and 648; HA-FIP3-4[A] - see Figure 2C) was also shown to modulate recycling endosome directionality, perhaps by regulating the interaction of FIP3 with dynein [14]. Since we and others have proposed that the accumulation of FIP3-containing vesicles in the midbody is a crucial facet of cytokinesis [6,18], we posited that the de/phosphorylation of FIP3 at these sites may control the interaction of FIP3-positive vesicles with the microtubule network and thus the distribution of FIP3 in the cell cycle. However, over-expression of HA-FIP3 (wild-type) and HA-FIP3-[4A] revealed that the 4A mutant did not affect cytokinesis, as revealed by quantification of the levels of binucleate cells (Additional file 1: Figure S1). The membrane/cytosol ratio of HA-FIP3 (wild-type) and HA-FIP3-[4A] in metaphase were similar (data not shown), consistent with these mutations not exerting a significant effect on the interaction of FIP3 with membranes in metaphase. Similarly, the cell cycle distribution of HA-FIP3-4A was indistinguishable from wild-type HA-FIP3 (Figure 5E). It will be necessary to examine the distribution of the corresponding Aspartate mutants to definitely rule out a role for phosphorylation/dephosphorylation of these residues, but the Alanine mutant employed here strongly suggests that phosphorylation/dephosphorylation of these sites may not play a crucial role in cytokinesis.

### Phosphorylation of FIP3 does not modulate GFP-FIP3 distribution or recycling endosome function in interphase cells

**Figure 6:**
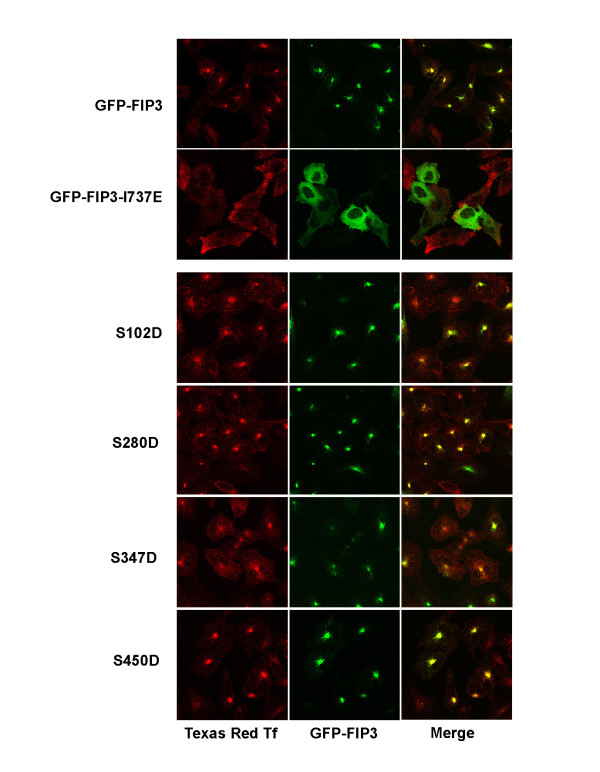
**Distribution of phosphomimetic mutants of FIP3 and their effect on transferrin uptake in interphase cells**. HeLa cells on glass coverslips were transiently transfected with GFP-FIP3 or the indicated mutants as described in *Methods*. 48 h later, cells were incubated for two hours in serum free D-MEM, transferred to media supplemented with 1 μg/ml human transferrin conjugated to Texas Red and incubated at 37°C for 30 minutes. Cells were fixed and the distribution of GFP-FIP3 (pseudo-coloured green) and Tf (pseudo-coloured red) analysed by confocal microscopy. Merged images were generated using the Zeiss confocal software. Shown are representative images for each mutant. Over 50 cells were analysed for each mutant at each condition, and each experiment was performed in triplicate.

The functional role of FIP3 is not confined to events in the cell cycle, but studies have revealed an important role for FIP3 in the control of recycling endosome distribution and function [8,13,19]. Previous studies have suggested that expression of GFP-FIP3 results in a clustering of FIP3 into a peri-nuclear region, concomitant with a clustering of recycling endosomes [7,19]. Expression of these mutant FIP3 species reveal that this clustering is unaffected by either the phospho-mimetic (S > D; Figure 6) or phospho-resistant mutations (not shown). This is in marked contrast to expression of GFP-FIP3-I737E, a Rab11-binding deficient mutant, which is predominantly cytosolic, consistent with a role for Rab11 in the recruitment of FIP3 to membranes [7,8]. Consistent with this, analysis of the uptake of fluorescently labelled transferrin revealed significant accumulation of label into the clustered GFP-FIP3 compartment, and that the corresponding S > D (Figure 6) and S > A (data not shown) mutants were without effect on transferrin accumulation. The data shown in Figure 6 are from cells exposed to labelled transferrin for 30 minutes; analysis of earlier time points (5 minutes) revealed no difference in accumulation between wild-type and phospho-mutants of FIP3 (data not shown). Thus, the phospho-acceptor sites identified here do not appear to modulate either the clustering of recycling endosomes induced by FIP3 over-expression, or the ability of cargo to traffic into these endosomes. Similar data has been reported for the HA-FIP3-[4A] mutant [14].

### FIP3 is a cell cycle-regulated phospho-protein

**Figure 7:**
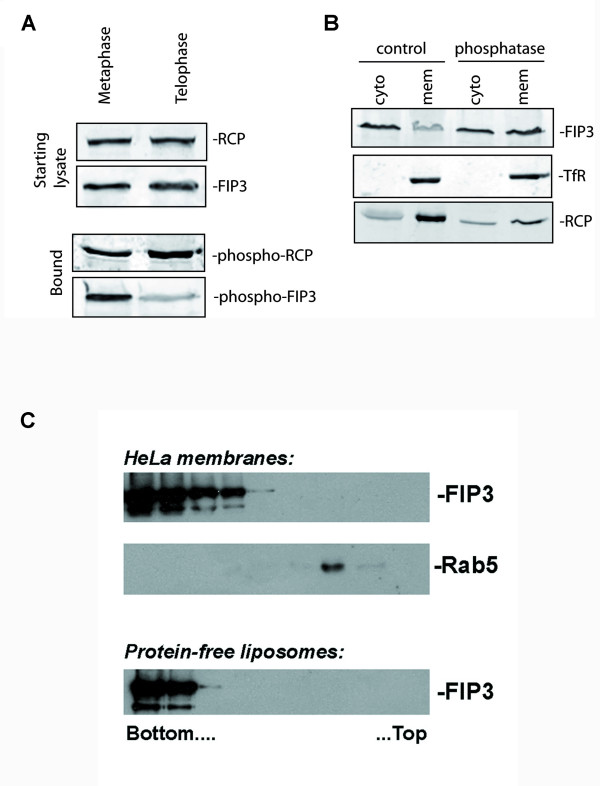
**Cytosol to membrane translocation of FIP3 occurs concomitantly with dephosphorylation**. **Panel A **Synchronized HeLa cells at prophase/metaphase (0 min) or telophase (70 min) were lysed in the presence of phosphatase inhibitors as described in *methods*. The lysates were then passed over a Phospho-Protein purification column (Qiagen). The column was washed to remove non-phosphorylated material, and phosphoproteins eluted. The amount of FIP3 and RCP were then analyzed by immunoblotting equal amounts of lysate or eluate, as shown. Data from a representative experiment is shown, repeated with similar results. **Panel B**. Synchronized HeLa cells were harvested immediately after release from nocodazole block. Half of the cell lysates were treated with alkaline phosphatase before fractionation of the lysates into cytosolic and membrane fractions. The levels of FIP3, TfR and RCP in all fractions were then analyzed by immunoblotting. Data from a representative experiment is shown, repeated three times. **Panel C**. Density gradient analysis of FIP3 association with HeLa membranes. Phosphatase-treated, recombinant FIP3 was incubated with either HeLa membranes or protein-free liposomes. These membranes (or vesicles) were then floated up through an Optiprep gradient. Fractions were collected and the distribution of proteins within the gradients was assayed by immunoblotting using the antibodies shown. The FIP3 sample incubated with HeLa membranes was blotted for Rab5, as a marker for endocytic membranes. The bottom and top of the Optiprep density gradient is marked. Data from a typical experiment, repeated with two different preparations of lipid vesicles and HeLa membranes is shown.

The data presented above suggest that the phospho-acceptor sites identified here (S-102, S-280, S-347 and S-450) play at best a minimal role in the control of FIP3 distribution of function. One explanation for this is that perhaps other, as yet unstudied, sites may be either more important or act cooperatively with those described here. Returning to our initial hypothesis that phosphorylation may represent a potential mechanism for the regulation of FIP3 cytosol/membrane ratio in the cell cycle, we used a phospho-protein affinity column to selectively bind phosphorylated proteins from HeLa extracts, isolated from prophase/metaphase arrested cells or from cells in telophase (Figure 7A). The phospho-affinity column retained FIP3 from prophase/metaphase arrested cells, but the extent of FIP3 binding was considerably reduced in telophase extracts. Such data suggest that FIP3 is phosphorylated in early stages of the cell cycle (metaphase) and dephosphorylated in later stages (telophase). As this corresponds to a movement of FIP3 from largely cytosolic (metaphase) to membrane associated (telophase), we tested this hypothesis by examining the membrane/cytosol distribution of FIP3 in metaphase homogenates, treated with or without phosphatase, and then subsequently separated into membrane and cytosol fractions. As revealed in Figure 7B, dephosphorylation of FIP3 consistently resulted in more FIP3 distributing to the membrane fraction. Such data support the hypothesis that FIP3 is a phosphoprotein. As a control for these experiments, we examined the distribution of another Rab11 effector, Rab Coupling Protein (RCP)/FIP1. The distribution of RCP/FIP1 between membrane and cytosolic fractions was not altered during the cell cycle (see Figure 1). Similarly, the levels of phosphorylated RCP/FIP1 did not differ between metaphase and telophase (Figure 7). Such data suggest that the cell cycle-induced changes in localization and phosphorylation are specific to FIP3. Although a modest change in membrane/cytosol ratio was observed for over-expressed GFP-FIP3-S102D, this did not approach the magnitude reported here for GFP-FIP3 (wild-type). Nonetheless, these data strongly support the notion that the cytosol to membrane translocation of FIP3 occurs concomitantly with dephosphorylation. In an attempt to determine whether this was a direct effect, we incubated recombinant FIP3 (pre-treated with alkaline phosphatase) with either artificial lipid vesicles or membranes from HeLa cells. We could not detect any direct interaction under these conditions (Figure 7C).

## Discussion

FIP3 is a Rab11 binding protein essential for the completion of abscission [8,9,20]. Previous work from our group and others has shown that FIP3 exhibits considerable changes in subcellular distribution throughout the cell cycle. Here, we have investigated the potential role of phosphorylation as a mechanism to control FIP3 distribution. We have shown that FIP3 is a cell cycle-regulated phosphoprotein (Figure 7), and that levels of phosphorylation of FIP3 decrease as cells move from anaphase into telophase (Figure 7). This dephosphorylation of FIP3 is accompanied by a shift of FIP3 from a largely cytosolic fraction to a membrane fraction (Figure 7), consistent with the observed distribution of GFP-FIP3 reported by us and others [8,21]. Using MS approaches we have identified four putative sites of FIP3 phosphorylation: S102, S280, S347 and S450 (Figure 1), extending the list of potential phospho-acceptor sites within FIP3 - see Figure 2C[14]. Of these, we show that phosphorylation of S102 is cell cycle regulated.

S102 lies within a consensus site for proline-directed kinases, including Cdk1 [17,22,23]. Since Cdk1 activity is tightly regulated during the cell cycle [3,17], we tested the hypothesis that Cdk1-cyclin B could phosphorylate this site *in vitro*. Figure 4 shows that recombinant Cdk1-cyclin B phosphorylated recombinant FIP3 *in vitro *solely on S102. Consistent with Cdk1-cyclin B kinase phosphorylating FIP3 in vivo, levels of pS102-FIP3 fall as cells progress from anaphase into telophase (Figure 3C, D). In an attempt to determine the functional significance of this phospho-acceptor site, we generated S102A and S102D mutants and expressed these in HeLa cells. Expression of GFP-FIP3-S102D but not GFP-FIP3-S102A resulted in a modest but reproducible increase in the number of binucleate cells, consistent with a defect in cytokinesis (Figure 5). However, this effect was significantly less than that observed upon expression of GFP-FIP3-I737E, a Rab11-binding defective mutant [8]. The molecular basis of the role of S-102 in cytokinesis remains to be established (see below).

We examined the functional consequences of S > A or S > D mutation at all of the sites identified here (S-102, S-280, S-347 and S-450). With the exception of S102D, over-expression of these mutants in HeLa cells had no effect on the numbers of binucleate cells or the distribution of FIP3 in the cell cycle (not shown). Of note, we also expressed all combinations of double, triple and quadruple Aspartate mutants. None of these exhibited significant increases in the numbers of binucleate cells (compared to S102D alone). Similarly, the interphase role of FIP3 in the spatial control of the recycling endosomal compartment and in transferrin recycling did not appear to be impaired by over-expression of any of the phospho-mutants (Figure 6). One exception to this concerns GFP-FIP3-S450A. We consistently observed that transfection of HeLa cells with GFP-FIP3-S450A led to significant cell death, with little, if any, immuno-reactive GFP-FIP3-S450A observed (not shown). By contrast, GFP-FIP3-S450D did not modulate cell growth, and its localisation was identical to that of GFP-FIP3. Interestingly, when double, triple or quadruple phospho-mutants were generated, any mutant containing S450A failed to express (data not shown). Such observations suggest that this residue may be involved in FIP3 stability, processing or folding, but further studies will be required to determine the basis of this.

More than 20 phospho-acceptor sites on FIP3 (and its fly homologue Nuclear Fallout) have recently been identified [14]. Phosphorylation of Nuf by IKK-ε has been shown to regulate the trafficking of Rab11-positive recycling endosomal-derived vesicles into and out of the fly bristle tip by coordinating its interaction with dynein [14,24]. Mutation of S-488, 538, 647 and 648 to A (HA-FIP3-4[A]) was also shown to modulate the ability of IKK-ε to control recycling endosome directionality [14]. We hypothesised that de/phosphorylation of FIP3 at these sites may control the interaction of FIP3-positive vesicles with the microtubule network and thus modulate the function and/or distribution of FIP3 in the cell cycle. However, over-expression of HA-FIP3 (wild-type) or HA-FIP3-[4A] did not affect cytokinesis as revealed by quantification of the levels of binucleate cells (Additional file 1: Figure S1). Similarly, the membrane/cytosol ratio of HA-FIP3 (wild-type) and HA-FIP3-[4A] in metaphase were similar (Additional file 1: Figure S1), consistent with these mutations not exerting a significant effect on the interaction of FIP3 with membranes. Similarly, the distribution of this mutant during the cell cycle was indistinguishable from the HA-tagged wild-type FIP3 (Figure 5).

### FIP3 is a cell cycle regulated phosphoprotein

The identification of a range of phospho-acceptor sites within FIP3 begs the question of the functional role of these sites. Given the significant redistribution of GFP-tagged FIP3 from cytosol to membranes (Figure 1) during the cell cycle and our observation that S-102 is dephosphorylated during progression from metaphase to telophase (Figure 3), we further explored whether phosphorylation of FIP3 is modulated during the cell cycle. To test this, we used a phospho-protein affinity column to selectively bind phosphorylated proteins from HeLa extracts, isolated from prophase/metaphase arrested cells or from cells in telophase (Figure 7A). The phospho-affinity column retained FIP3 from prophase/metaphase arrested cells, but the extent of FIP3 binding was considerably reduced in telophase extracts. Such data suggest that FIP3 is phosphorylated in early stages of the cell cycle (metaphase) and dephosphorylated in later stages (telophase). As this corresponds to a movement of FIP3 from largely cytosolic (metaphase) to membrane associated (telophase), we tested this hypothesis by examining the membrane/cytosol distribution of FIP3 in metaphase homogenates, treated with or without phosphatase, and then subsequently separated into membrane and cytosol fractions. As revealed in Figure 7B, dephosphorylation of FIP3 consistently resulted in more FIP3 distributing to the membrane fraction. Such data support the hypothesis that FIP3 is a phosphoprotein. As a control for these experiments, we examined the distribution of another Rab11 effector, Rab Coupling Protein (RCP)/FIP1. The distribution of RCP/FIP1 between membrane and cytosolic fractions was not altered during the cell cycle. Similarly, the levels of phosphorylated RCP/FIP1 did not differ between metaphase and telophase. Such data suggest that the cell cycle-induced changes in localization and phosphorylation are specific to FIP3.

These data suggest that dephosphorylation of FIP3 accompanies its redistribution from a cytosolic fraction to a membrane fraction. Several lines of argument suggest that dephosphorylation of FIP3 alone is unlikely to be sufficient to mediate this. Firstly, we and others have shown that FIP3 is recruited to membranes via interaction with Rab11 [8,9,25]. Indeed, inspection of the data in Figure 6 reveals that a FIP3 mutant (I737E) unable to bind Rab11 is cytosolic [8]. Hence, the interaction with Rab11 is likely to be the most important factor controlling FIP3 membrane distribution. Secondly, recombinant FIP3 does not bind either to phospholipid vesicles or to intracellular membranes isolated from HeLa cells (Figure 7C). Finally, neither S > D nor S > A mutants exhibited altered distribution of GFP-FIP3 throughout the cell cycle (Additional file 2: Figure 2 and data not shown). Collectively, these data suggest that although FIP3 is a cell cycle-regulated phosphoprotein, the consequences of phosphorylation are not primarily to control the localisation of FIP3. This is also consistent with the location of the phospho-sites within FIP3: all four sites studied here are located towards the N-terminal domain of FIP3, and are not within the coiled-coil domain which encompasses the sites of Rab11, Arf6 and Cyk-4 interaction (Figure 2C) [8,11]. It should be noted that FIP3 has multiple other potential phospho-acceptor sites, and over 20 such sites have been reported to be phosphorylated *in vitro *[14]. Some of these sites are located within the coiled-coil domain, and may modulate the interaction of FIP3 with other proteins or possibly control FIP3 homo-dimerisation. Mutation of the sites reported here did not in our hands modulate the distribution of dynein (not shown), nor affect the re-distribution of recycling endosomes characteristic of FIP3 over-expression (Figure 6). Similarly, traffic of cargo into and out of the clustered endosomes was not modulated by either S > A or S > D mutation of these sites singly (Figure 6) or collectively.

## Conclusions

Here we have shown that FIP3 is a cell cycle regulated phosphoprotein, and that FIP3 dephosphorylation occurs concomitantly with the shift from largely cytosolic to membrane bound observed as cells pass from metaphase to telophase. The phospho-acceptor sites studied here do not appear to control these changes. There are several possible explanations for this. First, the sites identified are not involved in this interaction, or that multiple sites of de/phosphorylation are involved. Alternatively, it is possible that multiple mechanisms of FIP3 targeting exist: this is consistent with studies showing that Rab11, Arf6 and Ect2 are all binding partners for FIP3, and all may contribute to FIP3 localisation. It is unlikely that a single site of phosphorylation may control all of these interactions. We favour a model in which the membrane association of FIP3 is driven mainly by interaction with Rab11 (consistent with other studies) and that dephosphorylation of FIP3 may represent a distinct regulatory mechanism, perhaps involving motor protein interactions resulting in the accumulation of FIP3 in midbodies. Further work will be required to resolve these models.

## Methods

### Antibodies and reagents

Anti-peptide antibodies were generated against a 9 amino acid synthetic peptide encompassing phospho-S102 as the central residue, and containing an additional C-terminal Cys residue for conjugation to keyhole limpet hemocyanin [NH_2_-GQLA(p)SPDAPC-COOH]. Conjugation of the peptide and immunisation of rabbits was performed by the Scottish Blood Transfusion Service. Phospho-selective antibodies were purified by a two step method. Total IgG was purified using a Protein-A sepharose column and dialysed extensively against PBS (137 mM NaCl, 2.7 mM KCl, 8.1 mM NaH_2_P0_4_, 1.76 mM KH_2_P0_4_, pH7.4). The phospho-specific antibodies were then affinity-purified over a column of the phospho-peptide coupled to Sulpho-link resin (Peirce), eluted in 20 mM Glycine pH 2.5, dialysed against PBS and snap-frozen prior to use.

Mouse anti-GAPDH and rabbit anti-GFP antibodies were purchased from Abcam plc. (UK). Rat anti-tubulin, clone YL1/2 antibody was purchased from Millipore (UK) Ltd. Alexa Fluor^® ^568 goat anti-rat IgG was purchased from Invitrogen (UK) Ltd. Anti-FIP3, Rab11, and other antibodies were as described in [7-9].

Lipofectamine™ 2000, Texas-red transferrin, Myelin basic protein (MBP) and DAPI (4, 6-diamidino-2-phenyindole dilactate) were from Invitrogen (UK). Cyclin B-CDK1 kinase was purchased from Millipore (UK) Ltd. Adenosine 5' triphosphate, ATP, (γ-32P) 3000 Ci/mmol (10 mCi/ml) was from PerkinElmer Life and Analytical Sciences, Italy. Phospho-protein affinity columns were from Qiagen.

### Recombinant FIP3 production

Full-length human FIP3 was tagged with an N-terminal 6-His tag followed by a TEV cleavage site by sub-cloning into the baculovirus transfer vector pVL1392. Co-transfection and amplification of recombinant baculoviruses was done by using BacPAK transfection reagents (BD Clontech, Palo Alto, CA), following the manufacturer's protocols. In brief, 1 × 10^6 ^Sf9 cells were seeded into a 6-well plate and Bacfectin-DNA mixture was added drop-wise. After 5 days the P1 viral stock was harvested and further amplified to P2 and P3 stages. For protein production, 1 l of Sf9 cells at 2 million cells/ml were infected with 2 ml of P3 viral stock (approximate multiplicity of infection: 0.5) and harvested after 65 h. Cells were lysed in 50 mM Tris buffer (pH 7.5) containing 300 mM NaCl, and the cleared lysate was loaded onto a Ni-NTA column. Eluted 6His-FIP3 was further purified using a S75 size exclusion chromatography column. Yields were typically 3-5 mg/l with an estimated purity of > 85%.

### Cell culture, transfection and imaging

Human cervical carcinoma HeLa cells were cultured in Dulbecco's Modified Eagle Medium (D-MEM) with 4.5 g/L glucose, supplemented with 10% (v/v) Foetal Calf Serum EU, 2 mM Glutamine, 100 Units/ml Penicillin and 100 μg/ml Streptomycin. HeLa cells stably expressing GFP-FIP3 were maintained in the same media containing 100 μg/ml Hygromycin B (Sigma-Aldrich Company Ltd., UK). Cells were transfected with plasmid DNA, using Lipofectamine™ 2000 following the manufacturer's instructions in antibiotic free media. The antibiotic-free growth media was changed 4-6 h after transfection and replaced with normal growth media. Cells were processed for transgene expression 24 to 72 h post-transfection. For time-lapse microscopy, cells were grown on collagen-coated coverslips and 24 h later viewed using a 20× lens in an environmental chamber at 5% CO_2_, 37°C.

### Cell synchronisation

HeLa cells were synchronized using thymidine/nocodazole block as described in [11]. HeLa cells were incubated for 16 h in the presence of 5 mM thymidine, then washed and incubated in fresh serum-supplemented media. After 8 h, 100 ng/ml of nocodazole was added and cells were incubated for another 16 h. Cells arrested in metaphase were then dislodged by gently tapping the side of the dish. Cells were collected and released from nocodazole mitotic block by washing in serum-supplemented media. After release from thymidine/nocodazole block, HeLa cells reach late telophase in approximately 3 h (see [11]).

### Immunofluorescence and transferrin trafficking

Cells were grown on 12-well plates containing ethanol-sterilised 13 mm diameter glass coverslips for 24 h prior to processing. Cells were fixed with 4% (w/v) ρ-formaldehyde (4% ρ-formaldehyde, 1 mM CaCl_2_, 1 mM MgCl_2 _in PBS) and incubated at room temperature for 20 min. ρ-formaldehyde was quenched in 30 mM NH_4_Cl and after washing in PBS, cells were permeabilised with 0.1% (v/v) Triton (in PBS) for 4 min. Cells were blocked in immunofluorescence (IF) buffer (PBS containing 0.2% (w/v) fish skin gelatin and 0.1% (v/v) goat serum) then incubated with primary antibody for 1 h at room temperature. After washing, cells were further incubated for 1 h with secondary antibodies diluted in IF buffer. For nuclear staining, DAPI was added to wash buffers as described [8]. Coverslips were mounted using Shandon Immu-Mount (Thermo Fisher Scientific). Coverslips were analysed using a 63X Zeiss oil immersion objective, on a Zeiss confocal microscope (Carl Zeiss, Germany) equipped with a Zeiss LSM5 Pascal instrument. An argon laser was used to excite 488 nm Alexa Fluor^® ^Dyes and GFP fusion proteins. Helium neon lasers were used to excite 568 nm Alexa Fluor^® ^Dyes. Zeiss Pascal software was used to collect images, which were saved as.mdb files.

For transferrin uptake assays, transfected cells were incubated for two hours in serum free D-MEM, then transferred to media supplemented with human transferrin conjugated to Texas Red (1 μg/ml) at 37°C in 5% (v/v) CO2 for 5 - 30 mins (see figure legends). Following incubation, cells were fixed with 4% ρ-formaldehyde as above and mounted onto glass slides for imaging [7].

### GFP-FIP3 mutant construction

GFP-FIP3 was that described in [8]. Mutations (S > A or S > D) were generated using a QuickChange kit (Stratagene) according to the manufacturer's instructions using Advantage^® ^GC Genomic LA Polymerase from Clontech (France). Some of the mutants were made by Dundee Cell Products Ltd. (Dundee, Scotland). Mutants were fully sequenced on both strands. The 4A-mutant of FIP3 was as described in [14].

### Subcellular fractionation and immunoblotting

To isolate cytosol and membrane fractions, synchronized mitotic HeLa cells were resuspended in 5 mM Hepes, pH 7.4 buffer and incubated for 10 minutes. Cells were then lysed using glass-glass homogenizer. Nuclei and debris were then sedimented at 3, 000 × g. The supernatant was then further centrifuged for 1.5 hours at 100, 000 × g to generate a cytosol fraction (supernatant) and a membrane fraction (pellet). The pellet was extracted with 20 mM Hepes, pH 7.4, containing 100 mM NaCl and 1% (v/v) Triton X-100. The solubilized membrane fraction (supernatant) was then sedimented for 1.5 hours at 100, 000 × g. To determine the relative levels of FIP3 in the cytosol and membrane fractions, 60 μg of fraction was separated on SDS/PAGE and immunoblotted with anti-FIP3, anti-TfR and anti-FIP1/RCP antibodies [8,26,27]. TfR, an integral membrane protein, was used as a control for the purity of fractions. FIP1/RCP, another member of Rab11-FIP family of proteins that is not required for cytokinesis, was used as negative control [8].

To isolate phosphorylated protein fractions, HeLa cells were lysed in 20 mM Tris HCl, pH7.4, containing 100 mM NaCl and 1% (v/v) Triton X-100. Lysates were then applied to PhosphoProtein purification column (Qiagen) and phosphorylated proteins purified according to manufacturer's instructions. Phospho-protein fractions were then separated by SDS/PAGE and immunoblotted with anti-FIP3 and anti-FIP1/RCP antibodies.

### Density gradient analysis of FIP3 association with membranes

HeLa membranes were prepared by passing HeLa cells through a ball-bearing homogeniser with a 14 μm clearance in 20 mM Hepes, 250 mM sucrose, 2.5 mM EDTA pH7.4 (HES buffer). After a 5 min centrifugation at 3000 × *g *to pellet nuclei, total membranes were sedimented at 100, 000 ×*g *for 30 min, and re-suspended in HES buffer at around 2 mg/ml. Protein-free liposomes were prepared exactly as described in [28]. For the experiment shown, 30 μl of HeLa membranes, or 30 μl of protein-free liposomes were incubated with 10 μM recombinant FIP3 (previously incubated with alkaline phosphatase) on ice for 90 min with occasional mixing. After this, samples were mixed with OptiPrep such that the final concentration was 40% (v/v) then overlaid in a centrifuge tube with 20% and 5% (v/v) layers. Samples were centrifuged for 4 h at 4°C at 85, 000 ×*g *and fractions were collected from the bottom of the tube, protein precipitated by tri-chloroacetic acid and analysed by SDS-PAGE and immunoblotting.

### *In vitro *phosphorylation assay

Reactions were set up in screw-capped micro-centrifuge tubes, as follows: 50 ng recombinant Cdk1/cyclin B was added to kinase assay buffer (50 mM Hepes pH 7.5, 10 mM MgCl_2_, 2 mM MnCl_2_, 0.2 mM DTT and 50 μM ATP.Mg^2+^) to produce a final volume of 50 μl, followed by 2 μg or 5 μg recombinant FIP3. 2 μg MBP was used as a positive control for kinase activity. To start the reaction, 6 μCi ATP-γ-32P was added to each tube and reactions were incubated in a water bath at 30°C for 30 min. 20 μl of Laemmli sample buffer (with 80 mM DTT) was added to each tube to stop the reaction. Samples were separated by SDS-PAGE, dried and exposed to Kodak X-ray film in a cassette with no intensifying screen at room temperature for 24 to 96 h.

### Identification of phospho-sites: In vivo phospho-site identification

Synchronized HeLa cell lysates from twenty 10-cm plates were harvested at prophase/metaphase (0 min after release from the nocodazole block). Cells were lysed in the presence of 1% Triton X-100 and phosphatase inhibitors, then incubated overnight with either 20 μg/ml lysate of affinity-purified goat anti-FIP3 antibodies or 20 μg/ml lysate of purified nonspecific goat IgG, followed by addition of 100 μl of protein A-sepharose per 1 ml of lysate. Beads were then washed, eluted with 1% SDS and separated on a 7-14% gradient acrylamide gel. The resulting gel was Coomassie stained and FIP3 band was isolated. Gel bands were cut and in-gel digested using trypsin and proteinase K. Peptides were extracted from the gel pieces with 0.1% TFA/60% acetonitrile and lyophilized. Dried peptide samples from each gel band were rehydrated and loaded onto a microcapillary column (100 mm inner diameter fused silica) packed with 15 cm Aqua C18 reverse-phase material and then placed in-line with an LTQ linear trap mass spectrometer. Peptides were eluted with 2 h mobile gradient of acetonitrile/0.1% formic acid. Tandem mass spectra were analyzed using Sequest using a human-mouse-rat database concatenated to a randomized human-mouse-rat database. DTASelect was used to reassemble identified peptides into proteins.

### Identification of sites of recombinant FIP3 phosphorylated *in vitro*

*In vitro *phosphorylation assays were performed on Baculovirus-expressed recombinant FIP3 using recombinant Cdk1/cyclin B as described above with the following modifications: 200 ng recombinant kinase (or buffer for control reactions) was incubated in 0.1 ml of kinase assay buffer supplemented with 100 μM ATP.Mg^2+^. 50 μg recombinant FIP3 was added to initiate the reaction which was then incubated at 30°C for 60 min. Protein was TCA precipitated, centrifuged at 23, 880 g 15 minutes at 4°C and separated via SDS-PAGE using NuPAGE^® ^reagents (Invitrogen (UK) Ltd.). The gel was stained with Brilliant Blue G-Colloidal Concentrate (Sigma-Aldrich Ltd., UK). FIP3 was excised from the gel and the phosphorylation site mapping of kinase phosphorylated FIP3 was carried out commercially by the FingerPrints Proteomic Facility at the University of Dundee (UK). This service uses a technique known as parent ion scanning (also known as Parents of -79) to detect phosphorylated peptides in conjunction with the generation of tandem MS data to identify the protein(s) in the solution to determine the sites of phosphorylation in the protein.

## Abbreviations

Cdk: Cyclin-dependent kinase; FIP3: Rab11-Family of Interacting Protein-3; GFP: Green Fluorescent Protein; RCP/FIP1: Rab Coupling Protein.

## Competing interests

The authors declare that they have no competing interests.

## Authors' contributions

LLC performed the experimental analysis of all FIP3 mutants, and contributed to or performed individually all figures except Figure 6. GS, CW, RP performed the experiments shown in Figure 1 and 6. JM, XY and MCM performed some of the experimental work, generated the anti-phospho antibodies, and prepared/purified recombinant FIP3. TO and SH generated further constructs and assisted with interpretation of data. LLC, JM, RP and GWG interpreted data. LLC, RP and GWG wrote the manuscript. Funding was obtained by RP, SH and GWG. All authors read and approved the final manuscript.

## Supplementary Material

Additional file 1 **Figure S1**. HA-FIP3-4A does not modulate cytokinesis. HeLa cells on glass coverslips were transiently transfected with HA-FIP3, or HA-FIP3-4A as described. After 48 h, cells were fixed and cells expressing HA-tagged FIP3 identified by immunostaining as described. In parallel, microtubules (anti-tubulin) and DNA (DAPI) were also stained. The fraction of cells expressing each FIP3 species that were binucleate was then determined and is plotted graphically in panel A. The data shown are from a representative experiment: over 200 GFP-positive cells were counted per condition. The experiment was repeated three times with similar results. Inset shows an anti-HA immunoblot of cell lysates to verify broadly similar expression levels of each construct. Click here for file

Additional file 2 **Figure S2**. GFP-FIP3 distribution in early and late telophase is not modulated by phosphorylation of S280, S347 or Ser 450. HeLa cells on glass coverslips were transiently transfected with GFP-FIP3 (pseudo-coloured green), or the indicated mutants as described. After 24 h, cells were fixed and immunostained with anti-tubulin (pseudo-coloured red) and the distribution of cells in telophase examined. Shown are cells in early or late telophase (cf. Figure 5). Data from a representative experiment, repeated 5 times are shown. Click here for file
