# Supplementary figures and images for: Rab11-FIP3 is a cell cycle-regulated phosphoprotein
Source: BMC Cell Biol. 2012 Mar 8;13:4. doi: 10.1186/1471-2121-13-4 (PMC3310825; doi:10.1186/1471-2121-13-4)

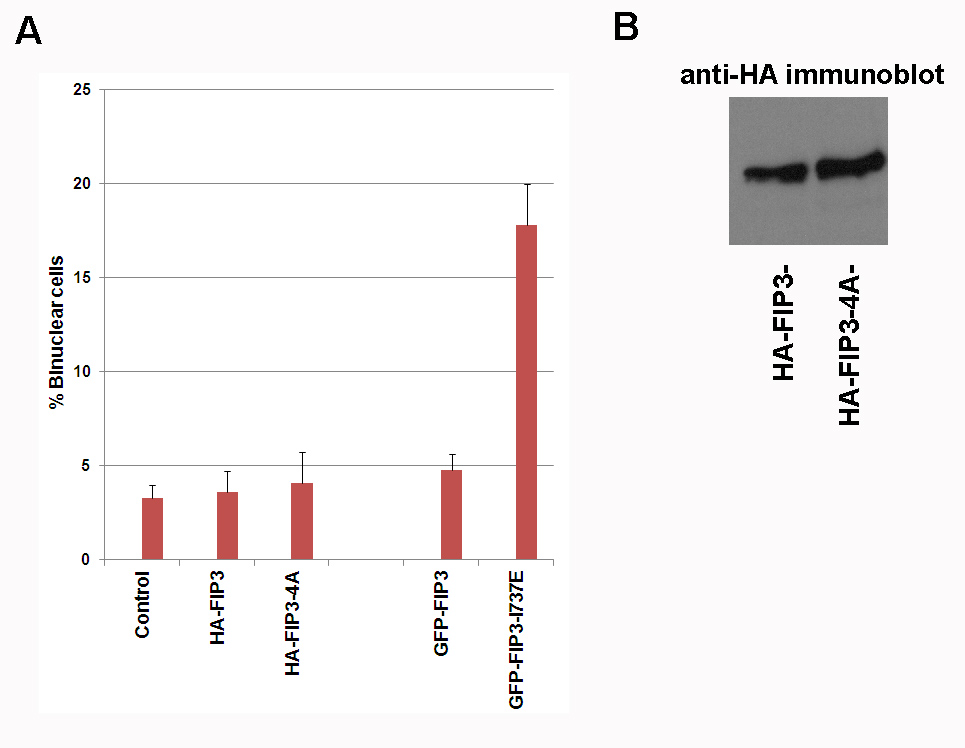

Supplement: Additional file 1 — Figure S1. HA-FIP3-4A does not modulate cytokinesis. HeLa cells on glass coverslips were transiently transfected with HA-FIP3, or HA-FIP3-4A as described. After 48 h, cells were fixed and cells expressing HA-tagged FIP3 identified by immunostaining as described. In parallel, microtubules (anti-tubulin) and DNA (DAPI) were also stained. The fraction of cells expressing each FIP3 species that were binucleate was then determined and is plotted graphically in panel A. The data shown are from a representative experiment: over 200 GFP-positive cells were counted per condition. The experiment was repeated three times with similar results. Inset shows an anti-HA immunoblot of cell lysates to verify broadly similar expression levels of each construct. [file 1471-2121-13-4-S1.JPEG]

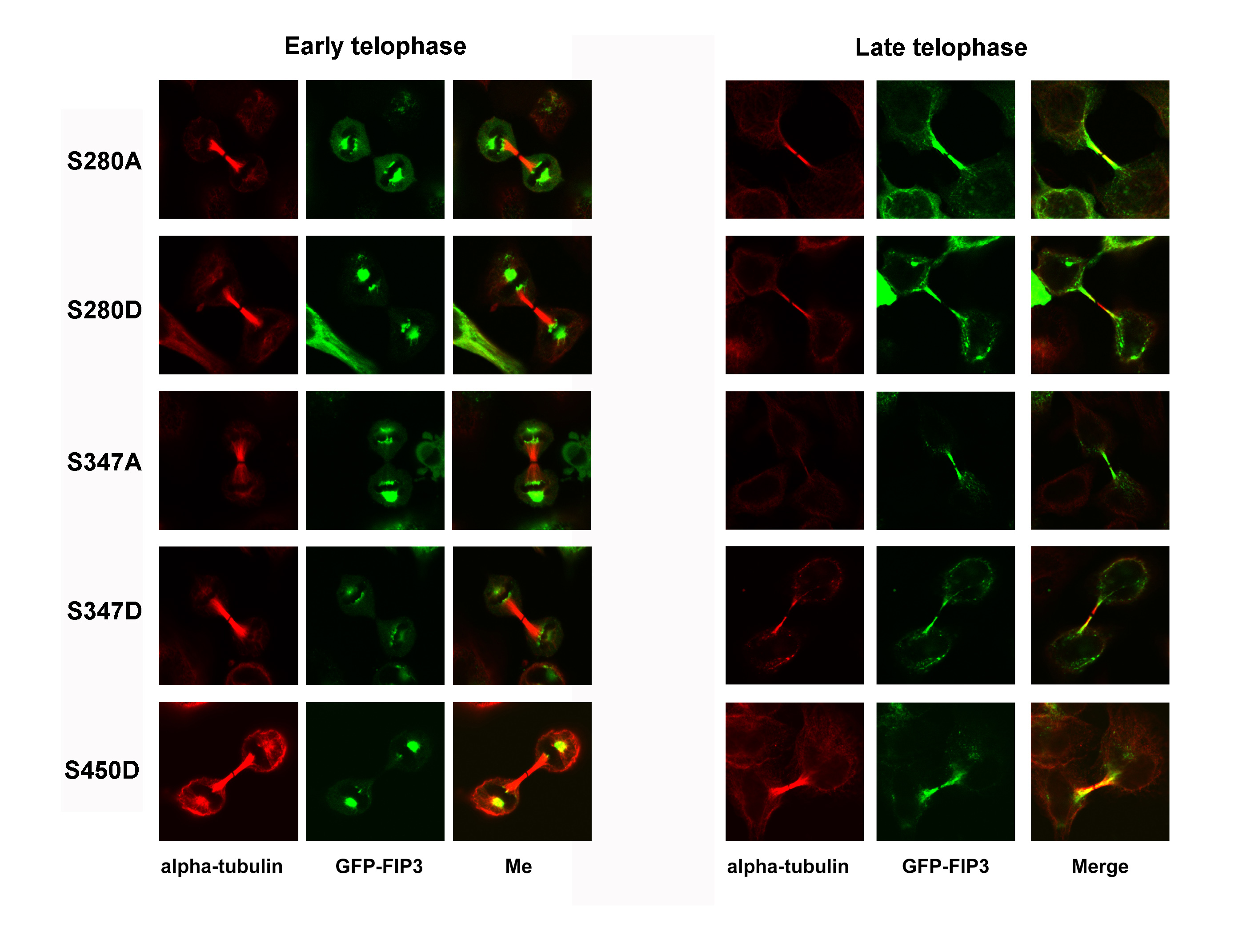

Supplement: Additional file 2 — Figure S2. GFP-FIP3 distribution in early and late telophase is not modulated by phosphorylation of S280, S347 or Ser 450. HeLa cells on glass coverslips were transiently transfected with GFP-FIP3 (pseudo-coloured green), or the indicated mutants as described. After 24 h, cells were fixed and immunostained with anti-tubulin (pseudo-coloured red) and the distribution of cells in telophase examined. Shown are cells in early or late telophase (cf. Figure 5). Data from a representative experiment, repeated 5 times are shown. [file 1471-2121-13-4-S2.JPEG]
